# Corrosion of an AZ31B Magnesium Alloy by Sulfate-Reducing Prokaryotes in a Mudflat Environment

**DOI:** 10.3390/microorganisms10050839

**Published:** 2022-04-19

**Authors:** Xiao Lan, Jie Zhang, Zaifeng Wang, Ruiyong Zhang, Wolfgang Sand, Liang Zhang, Jizhou Duan, Qingjun Zhu, Baorong Hou

**Affiliations:** 1 CAS Key Laboratory of Marine Environmental Corrosion and Bio-Fouling, Institute of Oceanology, Chinese Academy of Sciences, Qingdao 266071, China; lanxiao@qdio.ac.cn (X.L.); duanjz@qdio.ac.cn (J.D.); zhuqingjun@qdio.ac.cn (Q.Z.); brhou@qdio.ac.cn (B.H.); 2 Open Studio for Marine Corrosion and Protection, Pilot National Laboratory for Marine Science and Technology (Qingdao), Qingdao 266237, China; 3 University of Chinese Academy of Sciences, Beijing 100049, China; 4 Centre of Ocean Information Science and Technology, China National Offshore Oil Information Technology Co., Ltd., Beijing 100029, China; wangzf1@cnooc.com.cn (Z.W.); zhangliang47@cnooc.com.cn (L.Z.); 5 Aquatic Biotechnology, University of Duisburg-Essen, 45141 Essen, Germany; wolfgang.sand@uni-due.de; 6 Textile Pollution Controlling Engineering Center of Ministry of Environmental Protection, College of Environmental Science and Engineering, Donghua University, Shanghai 201620, China; 7 Institute of Biosciences, Freiberg University of Mining and Technology, 09599 Freiberg, Germany

**Keywords:** sulfate–reducing prokaryotes, mudflat environment, magnesium alloy, Microbiologically Influenced Corrosion, electrochemical impedance spectroscopy

## Abstract

To study the abnormal failure of magnesium anodes for buried pipelines in marine engineering in the unique environment of mudflats, a strain of a sulfate–reducing prokaryote (SRP) was isolated from pipe–laying soil, and identified as *Desulfovibrio* sp. HQM3. Weight–loss test, electrochemical measurements, SEM, EDS, XRD, and CLSM techniques were used to study the effect of corrosion on the AZ31B magnesium alloy. Under the influence of SRP, the magnesium alloy corroded severely at rates up to 1.31 mm/year in the mudflat environment. SRP accelerated corrosion by 0.3mm/year. Pitting occurred on the samples in both abiotic and biotic systems. The pitting depth reached 163.47 μm in the biotic system after 14 days. The main composition of a petal–like corrosion product was Mg(OH)_2_. The results show that a mudflat environment can lead to an accelerated corrosion of magnesium alloys.

## 1. Introduction

Due to the extensive laying of oil and gas pipelines, the corrosion protection in coastal mudflat areas is a challenging issue. These areas refer to the coastal zone flooded periodically by seawater, the so–called intertidal zone [1,2]. It is a complex environment with a three–phasic mixture of solids, liquid, and gas. It is more oxygen–rich than submarine soils, as the tides change periodically. It is a risky corrosive environment. Some studies believe that the corrosion of metals in the flat tidal environment mainly is resulting from the action of microorganisms and soil [3].

The annual direct costs caused by microbial corrosion in the world is about 30–50 billion U.S. dollars in marine environments. Therefore, Microbially Influenced Corrosion (MIC) has been paid great attention by scholars [4,5]. Sulfate–reducing prokaryotes (SRP) are typical corrosive microorganisms. Fathy et al. [6] believe that more than 75% of the corrosion of oil–producing wells and more than 50% of the corrosion of buried pipelines are caused by the activities of such microorganisms. In the mudflat environment microorganisms particularly tend to grow in the mudflat environment [7], which may result in localized corrosion accelerating the failure of metals [8].

Magnesium alloys are widely used for the cathodic protection of pipelines as sacrificial anodes [9,10,11], but severe self–corrosion limits their application in coastal mudflat environments [12,13,14]. Some studies have argued that a sustained expansion of local corrosion of magnesium anodes leads to spalling off of anode particles [15]. Other theories attribute it to the surface of a magnesium anode covered with corrosion products and uncovered parts causing galvanic corrosion [16], or the presence of a second phase or impurity elements resulting in micro–galvanic corrosion [17]. MIC is an important corrosion mechanism for materials in a wide variety of industries. The mechanism of MIC for magnesium alloys is not yet fully clear. One study found that SRP accelerate the micro–electro–couple corrosion on the surface of magnesium alloy by cathodic depolarization [18]. Starosvetsky et al. found that magnesium–aluminum alloys suffered more severe corrosion than pure aluminum in the presence of SRP [19]. AZ91Ce alloy is susceptible to crystal boundary corrosion under SRP conditions [20]. Heat treatment significantly affected microgalvanic corrosion behaviour between cathodic β–Mg_17_Al_12_ phase and anodic α–Mg matrix and improved corrosion behaviour [21]. Still, another study concluded that magnesium has an intrinsic antimicrobial bactericidal ability [22,23,24]. Therefore, it is instructive to study the corrosion effect of SRP on magnesium alloys. Investigations on the MIC of magnesium alloys have been limited mostly to the culture medium, which does not effectively reflect practical applications.

Nowadays, most studies are performed with simulated soil solutions [25,26]. Liu et al. [27,28] reported that in the soil simulant, SRP promote corrosion of carbon steel, but the soil layer affects the adhesion of SRP to reduce the corrosion rate. However, these differ significantly from the actual environmental conditions. In soil, bacteria generally are found as separated microscopic colonies on the surface and inside the voids covered by a thin water film, rather than planktonic or in an aqueous environment [29]. Yang et al. reported on the synergistic effects of deposits and SRP on the corrosion of a carbon steel [8]. Therefore, the simulated soil environment is more reflective of the actual situation. In this work, we have studied the corrosion behavior of magnesium alloys in the ocean mudflat environment with SRP. The corrosion acceleration was analyzed by using electrochemical measurements, Confocal laser scanning microscopy (CLSM), Scanning electron microscope (SEM), Energy dispersive spectroscopy (EDS), X-ray diffraction (XRD), and other methodologies.

## 2. Materials and Methods

### 2.1. Materials and Specimens

**Figure 1:**
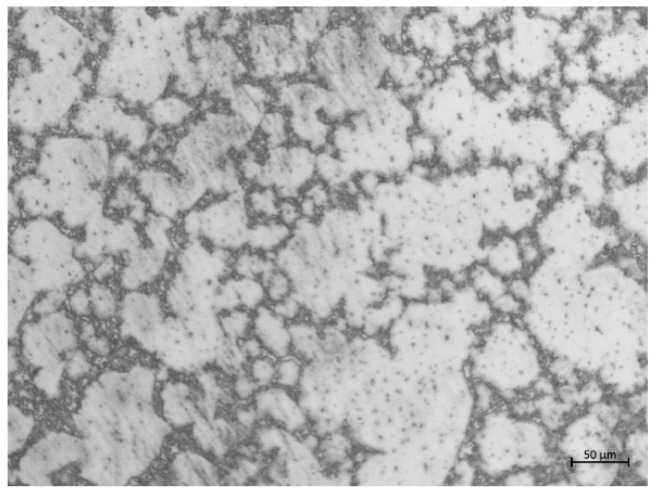
Image of AZ31B showing microstructure after treatment with 4% nital.

The material was an AZ31B magnesium alloy, supplied by Zibo Deyuan metal materials Co., Ltd. (Shandong, China). Its composition is given in Table 1. The specimens were polished mechanically by SiC paper up to a size of 5000 grit before tests. Figure 1. shows the optical microstructure of the specimen after etching with 4% nital (a solution of 4% nitric acid and alcohol). The specimens with dimensions of 10 mm × 10 mm × 3 mm were embedded in epoxy resin, leaving a free working area of 10 mm × 10 mm.

### 2.2. Bacterial Cultivation

**Figure 2:**
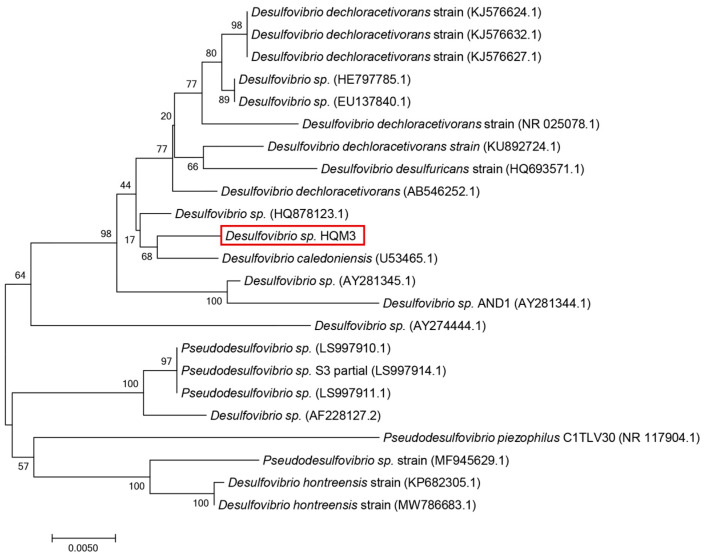
Phylogenetic position of *Desulfovibrio* sp. HQM3.

The SRP strain was identified as *Desulfovibrio* sp. HQM3. It was isolated from the tidal flat sediment near the pipeline laying on the west coast of Da Hengqin Island (113.45° E, 22.12° N) in Zhuhai, Guangdong Province, China. The 16S rRNA sequences have been deposited in the GenBank database with the accession number OK644303. Phylogenetic and evolutionary molecular analyses were conducted using MEGA version 7.0. The result is shown in Figure 2. The culture medium was composed of 0.5 g of KH_2_PO_4_, 1 g of NH_4_Cl, 0.06 g of CaCl_2_·6H_2_O, 0.06 g of MgSO_4_·7H_2_O, 6 mL of 70% sodium lactate, 1 g of yeast extract, 0.3 g of sodium citrate, and 1000 mL of filtered natural seawater (pH between 7.0 and 7.5). The fresh liquid culture medium was autoclaved at 121 °C for 30 min. The experimental tidal flats sediment was dried and sieved through a 1 mm aperture to filter out impurities such as plant roots. Then the sediment was autoclaved at 121 °C for 30 min and mixed with the modified medium at a ratio of 2:1 (note: ratio obtained by measuring the water content of the mud by the loss–in–weight method GB/T 39637-2020). The modified medium contained a 1% solution of bacteria (10^6^ cells/mL) in the exponential growth phase.

### 2.3. Corrosion Rate by Weight Loss

The specimens with a volume of 10 mm × 10 mm × 3 mm were used for weight loss tests. The experiment was divided into two cycles of 14 and 21 days in triplicate. Samples were immersed in simulated mudflat sediments with and without SRP. Before testing the samples were weighed on a high–precision balance. Finally, the samples were immersed in 1% AgNO_3_ + 15% CrO_3_ solution and heated to eliminate corrosion products. The samples were re–weighed according to the national standard (GB/T39637-2020). The corrosion rate was calculated as follows:

(1) $$\nu =8.76\times \frac{w_{0}-w_{1}}{AT\rho },$$

where ν is the average corrosion rate, mm/year; $w_{0}$ is the initial sample weight, g; $w_{1}$ is the weight of the sample after removing the corrosion products, g; A is the area of sample m^2^; T is the exposure time, hour; ρ is the density of the metal, g/cm^3^.

The relative corrosion rate of the samples is calculated as follows:

(2) $$v^{\prime }=v_{1}-v_{0},$$

$\;v^{\prime }$ is the relative corrosion rate, mm/year; $v_{1}$ is the corrosion rate of the sample within the SRP mudflat sediment, mm/year; $v_{0}$ is the corrosion rate of the sample without SRP mudflat sediment, mm/year.

### 2.4. Electrochemical Measurements

The electrochemical profile was measured using a Gamry 1000 potentiostat (Interface 1000, Gamry Instruments, Warminster, PA, USA); the samples were used as the working electrode (WE). A saturated calomel electrode (SCE) and a platinum sheet electrode were used as the reference electrode (RE) and counter electrode (CE). All EIS tests were carried out at open circuit potential (OCP). 10 mV sinus alternating amplitude signals with a frequency range from 10^5^ to 10^−2^ Hz curve fitting was carried out using ZsimpWin version 3.60. All measurements were carried out at 25 ± 2 °C.

### 2.5. Surface Analysis and Corrosion Product Analysis

After testing, the specimens were removed from the solution, immersed in a 4% (*w*/*w*) glutaraldehyde solution for 3 h, sequentially dehydrated with alcohol for 10 min at various concentrations (25%, 50%, 60%, 70%, 80%, 90% and 100% (*w*/*w*)), and dried by nitrogen blowing. A SEM JSM-7610F (JEOL Ltd., Tokyo, Japan) was used to visualize the morphology of the corrosion products and SRP on the surface of the specimens. The elemental composition of the corrosion products was assessed by an Ultra Dry EDS Detector (Thermo Fisher Scientific Inc., Waltham, MA, USA). In addition, the depth of the corrosion pits was measured by CLSM Lext OLS5000 (Olympus, Tokyo, Japan) after removing corrosion products. The corrosion product was gently scraped off the surface of the test piece with a razor blade. Element composition of the corrosion product was analyzed by X-ray diffractometry (XRD, Rigaku D/max-3C, Tokyo, Japan) with Cu Kα radiation. The XRD spectra were collected at angles between 5° and 80° at a rate of 10°/min.

## 3. Results and Discussion

### 3.1. Corrosion Rate of AZ31B Determined by Weight–Loss Measurement

**Figure 3:**
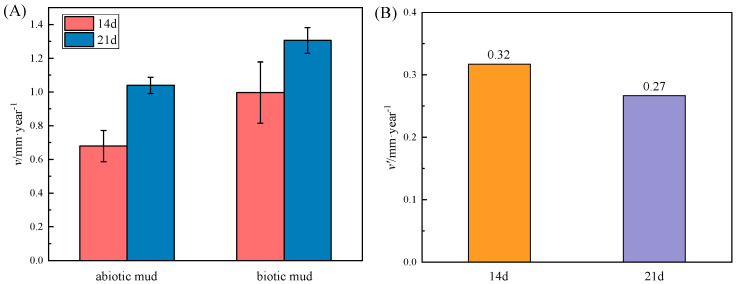
(**A**) Corrosion rate of AZ31B specimens in biotic and abiotic systems, (**B**) versus relative corrosion rate of SRP in mudflat environment.

As shown in Figure 3, after 14 days and 21 days of weight–loss testing, the corrosion in mud with SRP was more severe than in the abiotic assays, which shows that SRP–induced MIC is a significant factor in exacerbating the corrosion. The corrosion rate accelerates with time. In contrast, the effect of SRP became weaker after 21 days, which may be due to the depletion of nutrients in the reaction system.

### 3.2. Characterization of Corrosion Products on Specimen Surfaces

**Figure 4:**
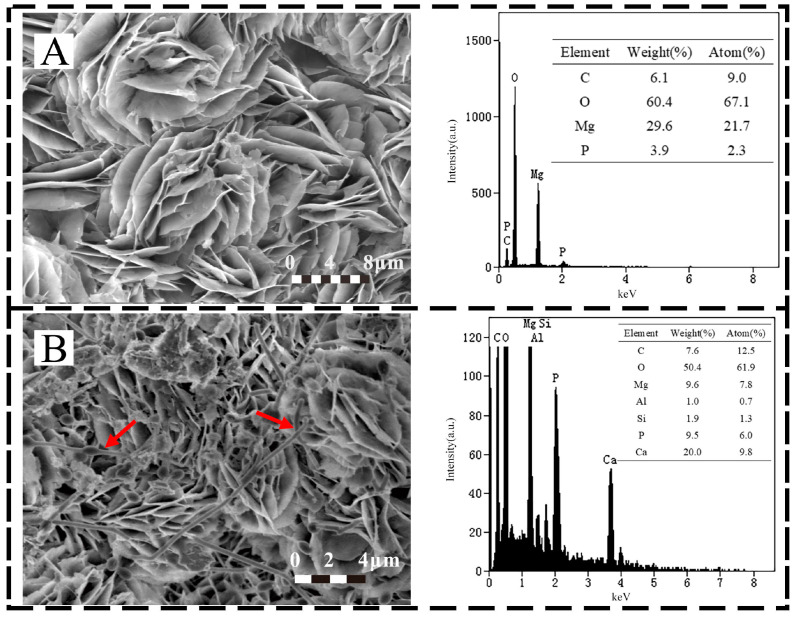
Selected SEM images and EDS analysis before removing the corrosion products on the AZ31B specimen after 14 days immersion under (**A**) abiotic mud (**B**) biotic mud. Arrows in B indicate cells.

SEM images and EDS results are shown in Figure 4. The SRP were enriched on the surface of the specimen and grew in the pores of corrosion products. Petal shapes were observed in the corrosion products of both specimens [30], and these were oriented nearly perpendicular to the specimen surface. SRP clearly were attached on the surface. At the same time, calcium deposition occurred on the surface of the biotic sample. As the dissolution of magnesium produces OH^−^ ions. The equation for the formation of calcium deposits is as follows [31]:

Ca^2+^ + HCO_3_^−^ + OH^−^ → CaCO_3_↓ + H_2_O (3)

This type of insulation product may be part of the reason for the abnormal failure of the sacrificial anode.

### 3.3. Pitting Morphology

**Figure 5:**
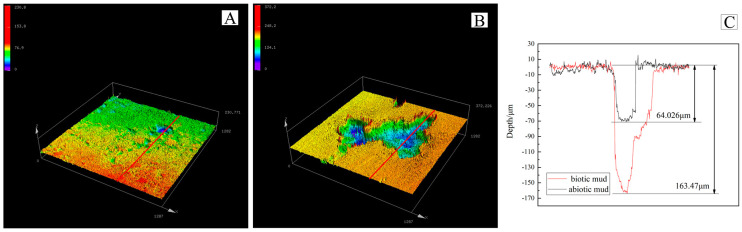
CLSM images of coupons immersed in different conditions for 14 days after removal of the surface products. (**A**) abiotic mud, (**B**) biotic mud, (**C**) comparison chart of maximum pit depth.

After removing the corrosion products and cleaning the coupon surfaces, the pitting morphology was visualized using CLSM Lext OLS5000 (Olympus, Tokyo, Japan). As shown in Figure 5, similar to the weight–loss testing the maximum corrosion pit depth for the specimen in the biotic mud reached 163.47 µm, 2.5 times deeper than that of the abiotic mud. Chen et al. reported that the variation of cathodic and anodic area ratios increases in exponential growth phase [32]. Therefore during the exponential growth phase the effective area for corrosion of the biological system may be smaller. The test for pitting depth increases the credibility of this conclusion. The activity of the SRP caused more severe pitting than observed for sterile systems [33].

### 3.4. Corrosion Product Analysis

**Figure 6:**
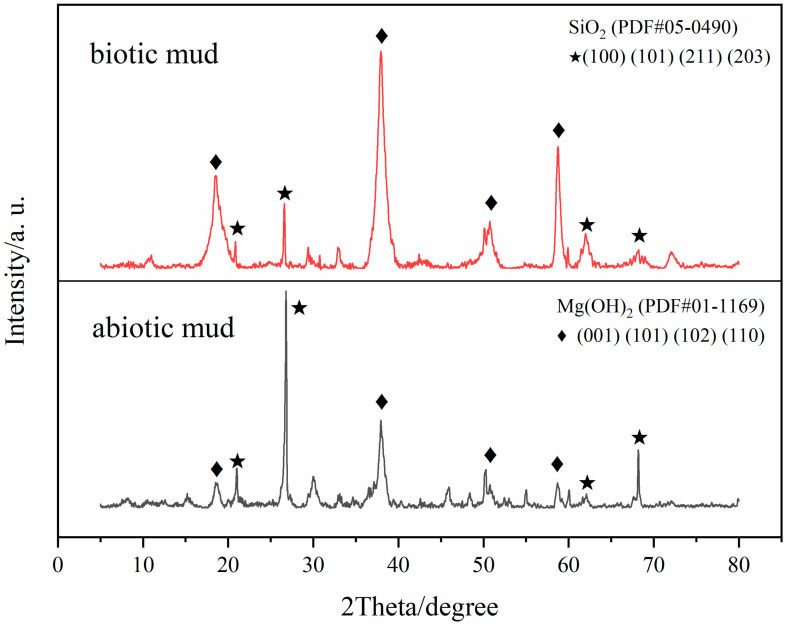
Selected XRD patterns of corrosion products on samples for 14 days.

As shown in Figure 6, the diffraction peak positions are consistent with Mg(OH)_2_ standard card PDF#01-1169 and SiO_2_ standard card PDF#05-0490. The XRD results show that the diffraction peaks correspond to the Mg(OH)_2_ (001) (101) (102) (110) crystal plane. The main component was Mg(OH)_2_, except for the residue of soil (SiO_2_) which had not been removed fully. The widely reported corrosion product is brucite [34,35], which would be formed based on the following reactions:

The anodic reaction:

Mg → Mg^2+^ + 2e^−^ (4a)

The cathodic reaction:

2H_2_O + 2e^−^ → H_2_ + 2OH^−^ (4b)

The product formation:

2Mg^2+^ + 4OH^−^ → Mg(OH)_2_ (5)

### 3.5. OCP Measurements

**Figure 7:**
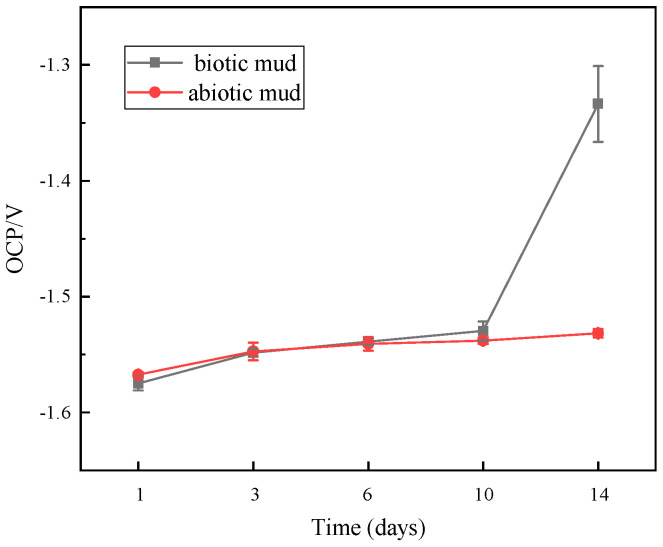
Time dependence of OCP under different corrosion conditions.

The open circuit potential can indicate the thermodynamic tendency of a metal to corrode [36]. A low OCP indicates a high thermodynamic tendency to rust. A high OCP can indicate a more passivated state or a low tendency to lose electrons [37]. The open circuit potential measurements are shown in Figure 7. As a function of immersion time the change in OCP can infer the formation of corrosion products on the AZ31B sample [38]. In the SRP system the OCP drifted towards the noble side 45 mV from −1.5747 V (vs. SCE) in the first 10 days and accelerated to drift 196 mV in the last four days to a final value of −1.3336 mV. The OCP drifted slowly from −1.5674 V to −1.5315 V (vs. SCE) in 14 days in the sterile system with a gradual stabilization. During the first 10 d the SRP concentration was at a low level and had little effect on the electrodes. Therefore, there was no significant difference. The accumulation of corrosion products was slightly faster with the sterile system. From day 10 onwards a significant positive shift in the SRP system occurred, which may be related to the accumulation of corrosion products, the growth of the bacteria, and the formation of biofilm [39,40]. The formation of biofilms and the growth of the bacteria will lead to accelerated corrosion. The corrosion rate of specimen in biotic system was higher than abiotic system in the weight–loss measurements in same time.

### 3.6. EIS Results

**Figure 8:**
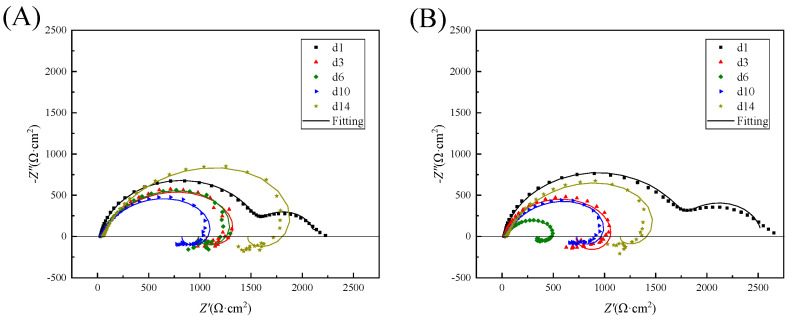
Nyquist plots of AZ31B magnesium alloy in different corrosion conditions recorded at different times. (**A**) abiotic mud, (**B**) biotic mud.

**Figure 9:**
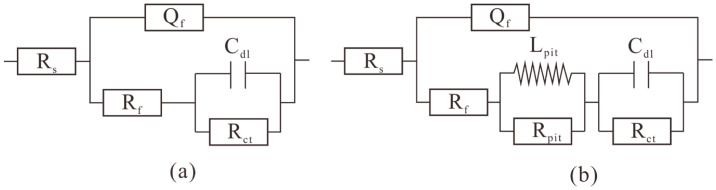
Equivalent circuit used to fit the EIS data in Figure 8. (**a**) d1; (**b**) d3–d14.

EIS is widely used to investigate the electrochemical processes that occur at the metal/solution interface. The electrochemical impedance spectra of magnesium alloys in beach mud with and without SRP were studied over 14 days. The results have been analyzed using ZsimpWin version 3.50., using the chi–square χ^2^ of the measured and fitted data to determine the quality of the fit of the equivalent circuit to ensure it was 10^−3^. The Nyquist diagram in Figure 8 shows that the impedance spectra in the low and high–frequency regions in the initial phase of both systems consist of one capacitive arc each, which corresponds to uniform corrosion [41]. The subsequent disappearance of the second capacitive arc corresponds to biofilm formation with the onset of submembrane pitting [42]. From day 3 on there existed a capacitive and an inductive arc. The radius of the capacitive arc in the high–frequency region decreases and then increases with time, which indicates that the corrosion rate of the test piece increased and then decreased [43,44]. As the surface product film became thick in the later stages of corrosion, a charge transfer became progressively more difficult, thus slowing down the corrosion rate. At the same time, the impedance arc radii of the SRP systems were all smaller than those of the sterile systems. It suggests that the SRP in the corrosion product layer facilitated corrosion. Charge transfer became progressively more difficult, leading to an increase in the radius of the capacitive impedance arc. In the low–frequency range, the appearance of inductive loops is generally attributed to Mg(OH)^+^ or Mg(OH)_2_ in the adsorbed state [45]. The changes in the electrochemical parameters are shown in Table 2 and Table 3. The equivalent circuit model R(Q(R(CR))) and R(Q(R(LR)(CR))) were employed for the fitting of the results. The equivalent circuit model [46] is shown in Figure 9. R_s_ denotes the solution resistance and Q_f_ denotes the surface layer capacitance. The impedance value of the constant phase angle component Q is described as follows: Z_CPE_ = Y_0_^−1^(jω)^−n^, where 0 < n < 1, Y_0_, and n parameters can be used to reflect the variation of the bilayer capacitance. R_f_ indicates the surface layer resistance, The inductive element L_pit_ and resistance R_pit_ in parallel constitute, respectively, the corrosion hole inductance and corrosion hole resistance; C_dl_ indicates the double electric layer capacitance; and R_ct_ indicates the charge transfer resistance. The R_ct_, and the test piece corrosion resistance are proportional [47]; the smaller the R_ct_ value, the greater the corrosion rate. The value of R_ct_ at day 1 in the SRP system higher than in the sterile system. This is due to the formation of a biofilm that temporarily reduces the corrosion rate of the specimen more than the reduced R_ct_ at d3–d15 than in the sterile system. It suggests that the occurrence of MIC caused the accelerated corrosion of the specimen.

### 3.7. Discussion of Mechanism

Summarizing the results of the electrochemical measurements and corrosion product analyses, the possible corrosion mechanisms of the alloy AZ31B incubated for 14 days in mudflats are as follows:

In the abiotic system, according to Song’s study [48] magnesium thermodynamically corrodes with the production of soluble ions. There is a potential passivation zone at high pH. In this study, however, the passivation zone was not reached. Magnesium suffers from severe self–corrosion and the overall reaction equation for corrosion is as follows [49]:

Mg + 2H_2_O → Mg^2+^ + 2OH^−^ + H_2_ → Mg(OH)_2_ + H_2_ (6)

**Figure 10:**
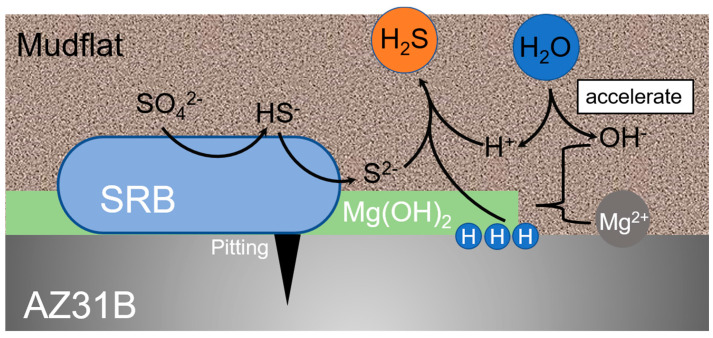
Schematic diagram of the mechanism, how SRP promote magnesium corrosion.

In the biotic system the role of the SRP cannot be ignored. The actual reaction steps of the MIC mechanism are complex, but there is no doubt that the oxidation of metals and the reduction of sulfate and many intermediates are involved in the MIC process [50]. As shown in Figure 10, the vital activity of SRP leads to the reduction of sulfate (as in Equation (7a,b)), consuming H^+^ from the system. It leads to an increase in the pH of the system [51]. This promotes hydrolysis, the formation of corrosion products and causes an accelerated corrosion of magnesium.

SO_4_^2−^ + 9H^+^ + 8e^−^ → HS^−^ + 4H_2_O (7a)

2HS^−^ + 2H^+^ → 2H_2_S (7b)

Magnesium with SRP undergoes the following reactions:

The anodic reaction:

Mg → Mg^2+^ + 2e^−^ (8)

The cathodic reaction:

H^+^ + e^−^ → [H] (9)

The cathodic depolarisation theory [52,53] suggests that the SRP consume hydrogen and, thus, promote magnesium corrosion. Environmental changes are less dramatic in a mudflat environment than in seawater. Corrosion products are more likely to remain on the surface of the material. Meanwhile, SRP consumes organic molecules on surface of the sample and produces extracellular polymers, organic acids and other metabolites [54,55]. These corrosive metabolites promote the dissolution of the metals. Biocatalytic cathodic sulfate reduction (BCSR) theory [56,57] suggests that SRP in the underlying biofilm have difficulty in obtaining a carbon source from system and must gain electrons by corroding the metal, leading to accelerated corrosion.

Many studies have shown that the corrosion of magnesium by SRP is pitting–based [20,33]. And it will further develop to the grain boundary and the second phase. A study reported that SRP stabilize a galvanic couple between a small anode and a large cathode which were initially of identical size [58]. Chen et al. [32] reported that the variation of cathodic and anodic area ratios in SRP media increases in exponential growth phase, maintains stable value in stationary phase. Hence, we suppose that SRP promote pitting during the exponential growth phase and maintain the cathodic and anodic area ratios during the stationary phase. They prevent the anode and cathode regions shifting over time. In general, severe localized corrosion carries a greater risk in actual application.

## 4. Conclusions

In this paper, the corrosion behavior of Mg caused by SRP in a mudflat environment has been investigated with the AZ31B Mg alloy for 21 days in mudflat with or without SRP. The following conclusions can be drawn:

1. The corrosion of the magnesium alloy in the biotic mudflat environment is severe with a corrosion rate of 1.31 mm/year, SRP contribute around 0.3 mm/year. The pitting depth reached 163.47 μm with Mg(OH)_2_ as the main component of the corrosion products.
2. Weight loss and electrochemical tests have shown that SRP in mudflat environment have a catalytic effect on the corrosion with uniform corrosion occurring first followed by localized corrosion such as severe pitting.
3. SRP accelerate the corrosion of magnesium sacrificial anodes and are one of the causes of the abnormal failure.

## Figures and Tables

**Table 1:** Chemical composition (in wt.%) of AZ31B.

Al	Si	Ca	Zn	Fe	Be	Mn	Cu	Mg
3.19	0.02	0.04	0.81	0.005	0.1	0.334	0.05	Bal

**Table 2:** Fitted electrochemical parameters for AZ31B magnesium alloy after immersion in biotic mudflat.

Time (Day)	R_s_ (Ω·cm^2^)	Q_f_ (F/cm^2^)	n	R_f_ (Ω·cm^2^)	L_pit_ (H·cm^2^)	R_pit_ (Ω·cm^2^)	C_dl_ (F/cm^2^)	R_ct_ (Ω·cm^2^)
1	19.3	2.70 × 10^−5^	8.96 × 10^−1^	1.82 × 10^3^	\	\	3.04 × 10^−3^	696
3	20.6	2.88 × 10^−5^	8.58 × 10^−1^	1.15 × 10^−22^	134	428	2.56 × 10^−6^	701
6	31.1	6.89 × 10^−5^	7.81 × 10^−1^	2.21 × 10^−3^	32.0	215	5.02 × 10^−6^	331
10	34.1	7.43 × 10^−5^	7.69 × 10^−1^	3.72 × 10^−3^	79.4	619	4.16 × 10^−6^	700
14	41.0	6.73 × 10^−5^	7.71 × 10^−1^	6.18 × 10^−8^	147	907	3.06 × 10^−6^	1120

**Table 3:** Fitted electrochemical parameters for AZ31B magnesium alloy after immersion in abiotic mudflat.

Time (Day)	R_s_ (Ω·cm^2^)	Q_f_ (F/cm^2^)	n	R_f_ (Ω·cm^2^)	L_pit_ (H·cm^2^)	R_pit_ (Ω·cm^2^)	C_dl_ (F/cm^2^)	R_ct_ (Ω·cm^2^)
1	28.6	2.20 × 10^−5^	9.04 × 10^−1^	1.58 × 10^3^	\	\	3.19 × 10^−3^	509
3	32.7	3.82 × 10^−5^	7.65 × 10^−1^	1.42 × 10^−3^	89.3	543	2.93 × 10^−6^	1030
6	38.5	5.18 × 10^−5^	7.82 × 10^−1^	2.49 × 10^−3^	98.2	700	2.44 × 10^−6^	951
10	33.7	5.11 × 10^−5^	7.88 × 10^−1^	1.48 × 10^−3^	90.7	520	3.63 × 10^−6^	793
14	52.7	5.57 × 10^−5^	7.81 ×10^−1^	1.00 × 10^−2^	212	1120	2.52 × 10^−6^	1420

## Data Availability

Not applicable.
